# Supplementary material for: Venomix: a simple bioinformatic pipeline for identifying and characterizing toxin gene candidates from transcriptomic data
Source: PeerJ. 2018 Jul 31;6:e5361. doi: 10.7717/peerj.5361 (PMC6074769; doi:10.7717/peerj.5361)
Supplement: Supplemental Information 3 [file peerj-06-5361-s003.gz › FinalOutput_E-20/Venom_peptide_isomerase_heavy_chain_2/finaltree.pdf]

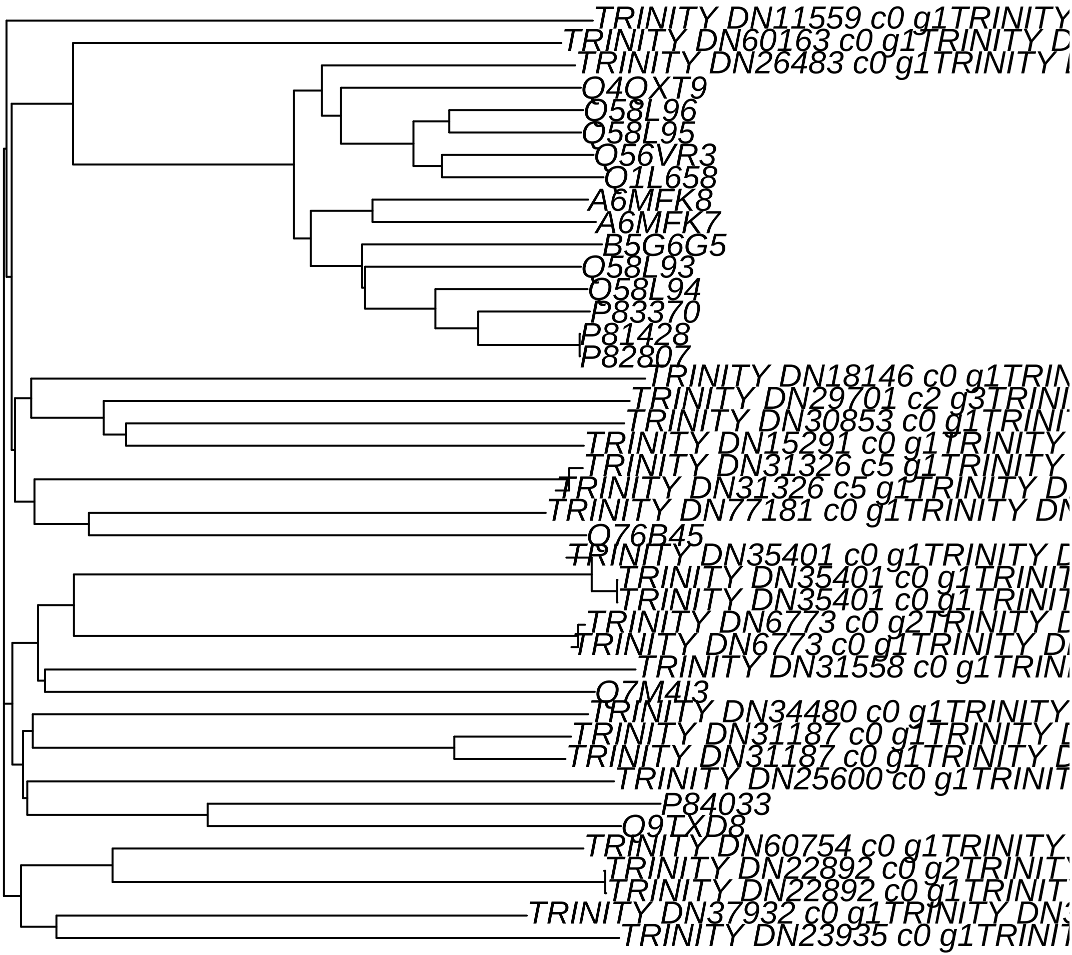

TRINITY DN11559 c0 g1TRINITY
TRINITY DN60163 c0 g1TRINITY D
TRINITY DN26483 c0 g1TRINITY
Q4QXT9
Q58L96
Q58L95
Q56VR3
Q1L658
A6MFK8
A6MFK7
B5G6G5
Q58L93
Q58L94
P83370
P81428
P82807
TRINITY DN18146 c0 g1TRIN
TRINITY DN29701 c2 g3TRINI
TRINITY DN30853 c0 g1TRINI
TRINITY DN15291 c0 g1TRINITY
TRINITY DN31326 c5 g1TRINITY
TRINITY DN31326 c5 g1TRINITY D
TRINITY DN77181 c0 g1TRINITY DN
Q76B45
TRINITY DN35401 c0 g1TRINITY D
TRINITY DN35401 c0 g1TRINIT
TRINITY DN35401 c0 g1TRINIT
TRINITY DN6773 c0 g2TRINITY D
TRINITY DN6773 c0 g1TRINITY D
TRINITY DN31558 c0 g1TRINI
Q7M4I3
TRINITY DN34480 c0 g1TRINITY
TRINITY DN31187 c0 g1TRINITY D
TRINITY DN31187 c0 g1TRINITY D
TRINITY DN25600 c0 g1TRINIT
P84033
Q9TXD8
TRINITY DN60754 c0 g1TRINITY
TRINITY DN22892 c0 g2TRINITY
TRINITY DN22892 c0 g1TRINIT
TRINITY DN37932 c0 g1TRINITY DN3
TRINITY DN23935 c0 g1TRINIT
